# The ‘dance’ of life: visualizing metamorphosis during pupation in the blow fly *Calliphora vicina* by X-ray video imaging and micro-computed tomography

**DOI:** 10.1098/rsos.160699

**Published:** 2017-01-25

**Authors:** Martin J. R. Hall, Thomas J. Simonsen, Daniel Martín-Vega

**Affiliations:** 1 Department of Life Sciences, Natural History Museum, Cromwell Road, London SW7 5BD, UK; 2 Naturhistorisk Museum Aarhus, 8000 Aarhus C, Denmark

**Keywords:** pupariation, gas bubble, holometaboly, development, morphology, Calliphoridae

## Abstract

The dramatic metamorphosis from larva to adult of insect orders such as Diptera cannot usually be witnessed because it occurs within an opaque structure. For the cyclorrhaphous dipterans, such as blow flies, this structure is the puparium, formed from the larval cuticle. Here, we reveal metamorphosis within the puparium of a blow fly at higher temporal resolution than previously possible with two-dimensional time-lapse videos created using the X-ray within a micro-computed tomography scanner, imaging development at 1 min and 2 min intervals. Our studies confirm that the most profound morphological changes occur during just 0.5% of the intrapuparial period (approx. equivalent to 1.25 h at 24°C) and demonstrate the significant potential of this technique to complement other methods for the study of developmental changes, such as hormone control and gene expression. We hope this will stimulate a renewed interest among students and researchers in the study of morphology and its astonishing transformation engendered by metamorphosis.

## 1. Introduction

The complete metamorphosis, holometaboly, of insect orders such as Diptera (flies), Lepidoptera (butterflies, moths), Coleoptera (beetles) and Hymenoptera (bees, wasps) dramatically transforms an immature larval stage to an adult through a pupal stage [1–3]. The pupal case generally provides a solid, opaque surround to the pupa, which prevents direct observation of the changes that occur within. This is certainly the case with the so-called ‘higher’ Diptera [4], the monophyletic Cyclorrhapha such as blow flies (Diptera: Calliphoridae), in which the extraordinary metamorphosis from legless larva to winged adult takes place within the puparium, a brown ovaloid case formed from the cuticle of the third and last larval instar [5–8]. For some Cyclorrhapha, the puparium is semi-transparent and some details of metamorphosis can be viewed under a light microscope, especially if the puparium is wetted for observation (e.g. *Drosophila* [9]). However, similar observation of the metamorphosis of blow flies is only possible for short periods by destructive opening of the puparium to expose the contents [10], or by applying mineral oil or microscopy immersion oil which, under intense illumination, provides sufficient transparency to observe some features of metamorphosis [11,12]. The difficulty in visualizing changes that occur within the intact puparium was cited as a major impediment in our understanding of insect metamorphosis [13].

The possibility of observing metamorphosis by methods other than light microscopy was shown by Thévenard [14], who used cineradiography techniques to record blow fly development within the opaque puparium on standard 35 mm film at speeds up to 32 frames per second, however, without providing a timetable for events. Subsequently, the early stages of intrapuparial development of the tsetse fly were recorded using a micro-focal X-ray [15]. The potential for three-dimensional X-ray micro-computed tomography (µCT) to peer inside the puparium and reveal developmental events has been demonstrated more recently [16,17]. The value of µCT to image blow fly puparia was shown by a study that imaged ethanol-fixed puparia and their contents during each of the quarters of development [17]. Since then, morphological changes in selected structures during metamorphosis within the chrysalis of a living butterfly, the Painted Lady *Vanessa cardui*, were revealed for the first time, using µCT to image developmental changes [18]. Although the interval for time-lapse imaging was higher than that of the blow fly study, it was only daily at most, meaning that temporal resolution was limited. If metamorphosis proceeds at a regular rate, then such low temporal resolution would not be a major issue. However, if development is punctuated by periods of rapid change then a higher temporal resolution would be needed to improve our visualization and understanding of metamorphosis. Indeed, such rapid change was indicated in *V. cardui*, as an adult tracheal system clearly different from the larval tracheal system was already evident in a scan of the first day of pupal development [18].

Major changes at a cellular and molecular level occur throughout metamorphosis under the control of ecdysone, a steroid hormone, including apoptosis of larval tissues and histogenesis of adult tissues [13,19]. However, during a study to identify developmental changes within the puparia of blow flies at a time resolution of 10% of the intrapuparial period, we noted that the most significant visible changes occur within a period of around 1.25 h at 24°C, equivalent to just 0.5% of the total intrapuparial period. These changes, essentially the eversion of adult tissues, occur within the first 10–20% of the intrapuparial period and were not imaged by earlier µCT studies [17], but they were noted by Thévenard [14] and a similar development sequence has been described in the fruit fly *Drosophila melanogaster* [20]. In this developmental interval, larval–pupal apolysis is already complete and the cryptocephalic (hidden head) pupa, whose body more closely resembles that of the larva than of the adult, transforms completely into the phanerocephalic (visible head) pupa by eversion of head and extension of legs and wings. During the next developmental intervals, pupal–adult apolysis (i.e. the separation of the pupal cuticle from the adult epidermis) takes place and, thereafter, the resulting pharate adult will gradually complete its development until emergence. The major changes that take place in this final phase are the development of the adult brain and sensory system, the development of the wings and associated flight musculature, the development of the genitalia and the transformation of the larval digestive system into that of the adult. The terminology of intrapuparial development can be confusing and has recently been reviewed [21].

During the initial 15% period of pupariation, blow flies and other cyclorrhaphous Diptera develop a gradually expanding gas bubble within the middle of the larval tissues [9,15,20,22,23]. Exactly how the bubble develops is still unknown [24], but it is most likely due to the intake of air through the posterior spiracles although it could also involve cavitation/embolism due to negative pressure following water loss, as in plant xylem [25]. Support for the bubble's development by the intake of air comes from the observation that if puparia are denied access to air, by being placed underwater for example, then the bubble does not develop [9]. Additionally, the gas bubble's volume in tsetse flies almost exactly equals the amount of water lost in water vapour [15], while the rate of water loss of blow fly puparia is greatly reduced by blocking the anterior and posterior spiracles [12]. The bubble is crucial to facilitate the dramatic morphological changes that take place during pupation. Mutated forms of *D. melanogaster* which fail to displace the bubble are unable to complete the eversion of head and legs [26].

A significant number of studies using a large number of techniques have described and imaged metamorphosis through the semi-transparent puparium of *D. melanogaster*, indeed the gas bubble and its translocation can be used as a marker for mutations with deleterious effects on development [26–29]. However, very few studies have looked in such detail at metamorphosis in blow flies. The synanthropic blow fly *Calliphora vicina* is of particular societal importance: as a pest of foodstuffs [30], as an agent of human and animal disease [31–33] and, beneficially, as a forensic indicator [34]. Here, we reveal for the first time metamorphosis during pupation within the puparium of this blow fly with two-dimensional time-lapse videos created using the X-ray within a µCT scanner, imaging puparia at 1 min and 2 min intervals, a higher frequency than previously applied. The period of observation in the videos follows movement of the bubble from the interior of the larval tissues into the space between the inner wall of the puparium and outer surface of the pupa and shows eversion of the head, separation of thorax and abdomen and the extension of the legs and wings.

## 2. Material and methods

Adult *Calliphora vicina* were collected from the grounds of the Natural History Museum (NHM), London (51.4952° N, 0.1755° W), identified according to standard identification keys [35] and maintained in culture at 23°C (±2°C), with free access to milk powder, sugar and water. The flies were provided with 2 ml of pig blood once daily for 10 days and then provided with fresh pig liver as an oviposition medium. Following oviposition, the liver with eggs was placed into a plastic box (160 × 160 × 86 mm), containing an approximate 3 cm depth of autoclaved soil, which was put into an incubator under a controlled temperature and humidity (mean = 24.0°C, s.d. = ±0.8°C; mean = 57.1% RH, s.d. = ±2.6% RH).

Samples for imaging were collected from post-feeding third instar larvae of wild caught parents at a time when their transformation into an ovaloid shape was irreversible, with a smooth cuticular surface apparent [5,6]. These puparia were maintained at the same temperature and humidity as before until the time for their imaging.

X-ray scanning was undertaken using a Nikon Metrology HMX ST 225 system. The chamber temperature of the CT-scanner was 23°C. Single images were reconstructed from a set of 32 exposures of 0.5 s each with an X-ray beam of 110 kV and 203 µA, passed through a 0.1 mm aluminium filter.

Twenty-four hours after their pupariation, unwashed puparia (approx. 9.6 × 3.9 mm) were stuck by double-sided adhesive tape to the floor of a Petri dish, aligned by eye so that either their dorsal or a lateral surface was uppermost. They were placed approximately 2 mm apart, with 9–10 puparia per dish in two rows. The Petri dish was placed in front of the X-ray beam so that the rays passed through from dorsal to ventral surface or from one side to the other. The dish was positioned in the scanner on its side so that the anterior end of each puparium was at the top. Images were taken initially at 10–30 min intervals of all the puparia until it was clear that the gas bubble was about to move from its central position in one or more. The image was then magnified to focus on two puparia and the temporal resolution was increased to one image every minute for approximately 3.5 h. The video (electronic supplementary material, Movies S1) was cropped to show only the puparium in which the gas bubble moved first. The brightness of the raw composite images was increased by 30% using the linear ‘adjust brightness/contrast’ tool in Adobe Photoshop v. CS4 (Adobe Systems, Inc.) and spliced together into a video using ImageJ v. 1.49u [36]. While the emphasis of our discussion is on the 1 min interval video, a preliminary video was produced in the same way, but using an imaging interval of 2 min and focusing on seven puparia, with the resultant video cropped for symmetry to show six puparia (electronic supplementary material, Movies S2).

Three-dimensional µCT images were taken of a different batch of puparia, with development approximately equivalent to stages shown at the start and end of the video. Five pupae were collected at 6, 24 and 30 h after pupariation, killed in hot water, stained in 0.5 M iodine and scanned using the same µCT system (exposure, 500 ms; voltage, 110–130 kV; current, 100 µA). The resulting projections were reconstructed with a voxel size of 9.5 µm using CT-Pro 2.1 (Nikon Metrology, Tring, UK). Reconstructed slice stacks in the three principal planes (cross, horizontal and sagittal) were rendered and visualized for each specimen using VG Studio Max 2.2 (Volume Graphics GmbH, Heidelberg, Germany). Two-dimensional images are shown here of the most informative slices.

A puparium with gas bubble was reconstructed in three dimensions using SPIERS v. 2.20 [37] and false coloured to show the relationship of the gas bubble to the larval gut, the malphigian tubules and the dorsal tracheal trunks.

## 3. Results and discussion

**Figure 1.**
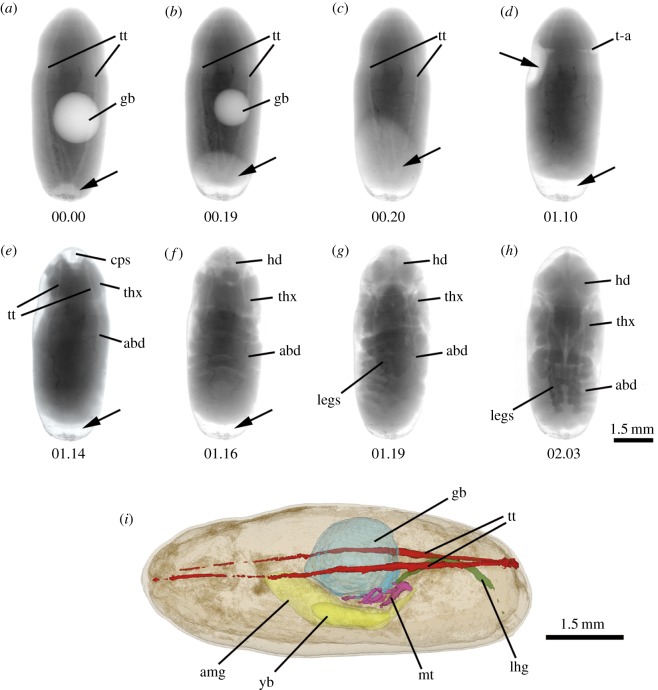
(*a*–*h*) Representative X-ray images captured from the video (S1) at the time indicated (h.min) after start of sequence (*a*, 00.00, corresponding to 28 h after pupariation). Details on the changes shown in each frame are provided in the text. The contents of the gas bubble began to notably move posteriorly after about 15 min, with the majority of gas movement from 17 to 20 min (*b*,*c*). Major morphological changes were completed after about 1 h 20 min of imaging (*g*). (*i*): False-colour three-dimensional reconstruction of puparium (approx. 9.6 × 3.9 mm) with gas bubble (blue) 24 h after the start of pupariation (abd, abdomen; amg, adult midgut; cps, cephalopharyngeal skeleton; gb, gas bubble; hd, head; lhg, apoptotic larval hindgut; mt, malphigian tubules; t-a, thorax--abdomen boundary; thx, thorax; tt, tracheal trunks; yb, yellow body. Arrows show lightened or cleared areas where the gas from the bubble is relocated).

The 1 min interval time-lapse video (figure 1 and electronic supplementary material, video, Movies S1) shows for the first time at a high temporal resolution the rapid and dramatic changes that take place during the short period of about 1.25 h at 24°C (equivalent to 0.5% of the intrapuparial period) in which metamorphosis inside the blow fly puparium is visibly at its most intense, resulting in the greatest morphological changes. The specimen imaged for the video was exposed to X-rays for a further 1.33 h beyond the end of our video due to the imaging of another specimen adjacent to it, so it was imaged at least 222 times. This high exposure to X-rays probably led to its non-emergence. Seven specimens imaged at 2 min intervals in a separate study (six shown in the electronic supplementary material, Movies S2) also did not emerge, but they pupated at the same rate as the specimen in the main video (table 1). However, most other specimens imaged fewer times in exploratory studies (at 5 min or greater intervals during pupation) did emerge successfully (16/18, 88.9%) and they pupated at the same overall rate as the specimens in our videos (data not included in table 1 because the more than or equal to 5 min intervals did not enable detailed timing of events). In addition, in a related study on the entire intrapuparial development of *C. vicina* maintained at the same temperatures as here, the numbers of puparia with gas bubbles at 26, 27, 28, 29 and 30 h after pupariation were, respectively, 9/9, 8/9, 7/10, 3/10 and 0/10 [38]. Thus, the major loss of the gas bubble was between hours 28 and 29, the same as we observed with the videoed specimens. Therefore, we are confident that the changes shown by the specimens in both videos (electronic supplementary material, Movies S1 and S2) accurately reflect natural pupation.

**Table 1.** Timing of major events following gas bubble shrinkage during the pupation of *Calliphora vicina* at 24°C, recorded from specimens X-ray imaged at either 1 min (*n* = 1) or 2 min (*n* = 7) intervals. As there was some variation in the time after pupariation when gas bubble shrinkage began, in order to compare specimens the time when the gas bubble began to visibly shrink within each pupa was designated as the start time of events, i.e. time zero.

description of event	*n*	mean time of event (minutes; ± s.d.)	range of times (minutes)
gas bubble visibly starts to shrink	8	0	0
gas bubble has disappeared from pupa	8	5.1 (± 1.5)	4–8
head starts to evert (thorax appears to ‘open’)	8	39.1 (± 11.2)	26–56
head completes eversion	7^a^	55.3 (± 9.6)	38–66
legs are fully extended	6^a^	75.3 (± 10.8)	54–83

^a^Note that the 2 min interval video ended before one pupa had completed head eversion and before one had completed full leg extension.

The main video (electronic supplementary material, Movies S1) opens with a view of the cryptocephalic pupa [21] containing a large and slightly off-centre internal gas bubble, with a small volume of gas external to the pupa at the posterior end of the puparium (figure 1*a*, 00.00). The paired longitudinal tracheal trunks, each with secondary branching, are visible extending from the posterior end, tapering towards the anterior end. The first clearly visible change in the video is the gradual shrinking of the gas bubble as its contents move from the centre of the pupa to the posterior end of the puparium, most gas movement occurring within the final 4 min of this process, forcing the cryptocephalic pupa anteriorly (figure 1*b*, 00.19). Although this has not been proven, the most probable route of gas exit is along the dorsal tracheal trunks [15], emerging from the posterior spiracles which become detached from the inner puparial wall [11]. The gas then moves from the posterior end anteriorly, between the pupa and inner surface of the puparium (figure 1*c*,*d*, 00.20–01.10). Towards the end of this stage, the boundary between thorax and abdomen becomes clear as an indentation (figure 1*d*, 01.10). Movement of the gas anteriorly forces the pupa posteriorly until a space is created at the anterior end of the puparium into which the head can be seen to evert (figure 1*e*–*g*, 01.14–01.19). The cephalopharyngeal skeleton of the third instar larva is most clearly visible during head eversion, attached to the anterior inner surface of the puparium (figure 1*e*, 01.14). The positions that the eyes will occupy are marked by the darker areas on each side of the head which indicate the sclerotized cornea (figure 1*g*). The legs can be seen extending posteriorly from their base on the ventral thorax of the now phanerocephalic pupa (figure 1*g*,*h*, 01.19–02.03). During pupation, the pupa is clearly in a dynamic state, owing to muscular contractions which withdraw the larval dorsal tracheal trunks [11], help move the gas bubble anteriorly [22] and evert and extend the adult tissues. A rise in oxygen consumption has been reported at this time corresponding to the period of physical activity that results in severance of the tracheae [12]. The large amount of movement of the pupa [10], including leg extension, combined with the jerkiness of the accelerated time-lapse makes it appear as if the pupa is ‘dancing’. The process of pupation of the single specimen imaged at 1 min intervals is mirrored in the six puparia imaged at 2 min intervals, but three of the latter were mounted with the left lateral surface uppermost to give a different perspective in which the wing and leg extensions are even more obvious.

**Figure 2.**
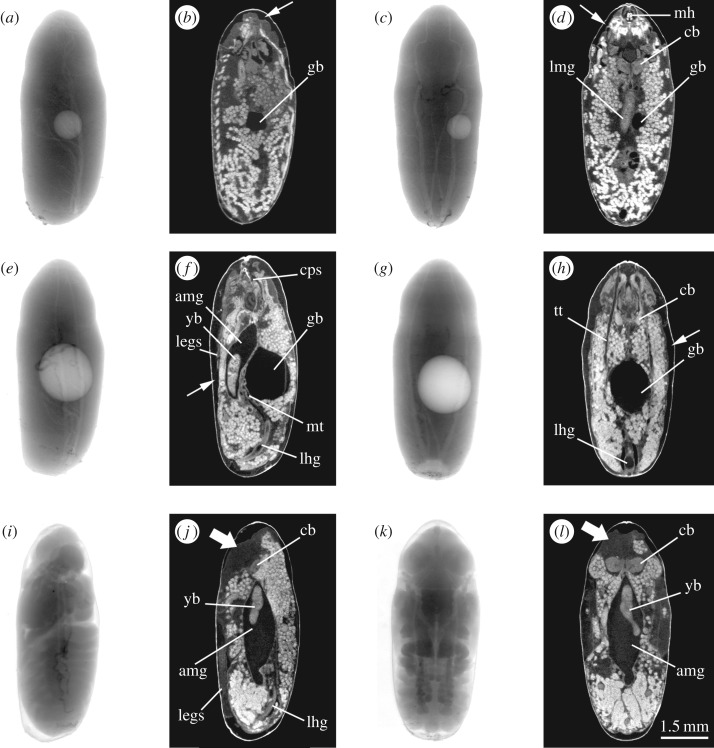
(*a*–*l*) Two-dimensional X-ray lateral (*a*,*e*,*i*) and dorsal (*c*,*g*,*k*) images and µCT-based virtual sagittal (*b*,*f*,*j*) and horizontal (*d*,*h*,*l*) sections through approximately the midline of puparia at approximately 6 (*a*–*d*), 24 (*e*–*h*) and 30 (*i*–*l*) hours old to demonstrate the major metamorphosis that occurs in around 0.5% of the intrapuparial period. Fine arrows (*b*,*d*,*f*) show where the pupal epidermis has detached from the inner wall of the puparium as larval–pupal apolysis proceeds. Thick arrows (*j*,*l*) show the hyaline appearance of the head, as fat bodies have still not dispersed throughout it (amg, adult midgut; cb, central brain; cps, cephalopharyngeal skeleton; gb, gas bubble; lhg, apoptotic larval hindgut; lmg, larval midgut; mh, mouthhooks of cephalopharyngeal skeleton; mt, malphigian tubules; tt, tracheal trunks; yb, yellow body).

Internally, the µCT virtual sections show how a small gas bubble is formed within 6 h of pupariation in the centre of the degenerating larval tissues. At the same time, larval–pupal apolysis (i.e. the separation of the epidermal cells of the pupa from the larval cuticle or puparium) has started in the anterior part of the prepupa, the stage between pupariation and larval–pupal apolysis (figure 2*a*–*d*). Electronic supplementary videos are provided of the full set of µCT sections for the three time points: 6, 24 and 30 h after pupariation (electronic supplementary material, Movies S3–S8). Just 24 h after pupariation, the larval–pupal apolysis is complete (leading to the cryptocephalic pupal stage) and the volume of the bubble is significantly increased (figure 2*e*–*h*). The imaginal discs of legs and wings evert (wings not visible in figure 2*f*,*h*, but see the electronic supplementary material, Movies S5 and S6) and the adult midgut can be observed as a closed, relatively small sack enclosing the yellow body (i.e. the apoptotic larval midgut cells; figure 2*e*–*h*). The three-dimensional virtual reconstruction (figure 1*i*) shows how the gas bubble is positioned between the main longitudinal tracheal trunks, occupying the central part of the body and displacing the adult midgut to the ventral side. Once the head is everted and the cryptocephalic pupa is transformed into the phanerocephalic pupa (figure 2*i–l*), the legs extend and the volume of the closed adult midgut increases, occupying the middle of the thorax and anterior portion of the abdomen, essentially replacing the gas bubble. At this time, the fat bodies (imaged as white globules) have still not dispersed throughout the head, giving it an ‘empty’ appearance (figure 2*j*,*l*).

Our study confirms the rapidity of the most profound morphological changes during the metamorphosis of cyclorrhaphous flies, supports the essential role of an intrapuparial gas bubble during these changes and demonstrates the value of the µCT technique to complement other methods for the study of developmental changes, such as hormone control [39–41] and gene expression [42,43]. The development series described here is overall similar to that of *D. melanogaster* [9,22]; however, the present *in toto* results using µCT are more detailed than previously published accounts for any fly species describing development of the entire pupa. While Aldaz *et al*.'s [44,45] fluorescence tomography studies of imaginal disc development in *Drosophila* did yield considerably higher spatial resolution, those studies focused on *ex vivo* development of only a single tissue component, i.e. the imaginal discs. Furthermore, the videos presented here enable visual appreciation of the speed of such complex changes for the first time. The time-lapse videos provide just a small indication of what will be possible in future to improve our visualization and understanding of insect metamorphosis. We hope that they will stimulate a renewed interest among students and researchers in the study of morphology and its astonishing transformation engendered by metamorphosis. Future work will use a µCT system with improved resolution enabling more detailed imaging of developmental changes within discrete organs like the brain. Lowering the X-ray energy should also enable us to image the metamorphosis events in living puparia in three dimensions, using analysis techniques similar to those used to successfully visualize the movement of flight muscles in living adult blow flies [46], giving a much better understanding of the disappearance of the gas bubble and, when applied to earlier periods in pupariation, its enigmatic development.

## Supplementary Material

More than five files

## Data accessibility

The videos reported in this paper can be accessed at the Dryad Digital Repository: http://dx.doi.org/10.5061/dryad.5p5cm [47].
