# Supplementary material for: The ‘dance’ of life: visualizing metamorphosis during pupation in the blow fly Calliphora vicina by X-ray video imaging and micro-computed tomography
Source: R Soc Open Sci. 2017 Jan 25;4(1):160699. doi: 10.1098/rsos.160699 (PMC5319339; doi:10.1098/rsos.160699)
Supplement: More than five files [file rsos160699supp1.docx]

**Movies S1**

Time-lapse X-ray video of pupation in *Calliphora vicina*, beginning at 28 hours after pupariation (00:00, h:min). Images are displayed at one frame per second, each image captured at intervals of one-minute (i.e. 60 x natural speed). The pupa was imaged with the dorsal surface uppermost and its head at the top of the video. See text and Figure 1 for further explanation.

**Movies S2**

Time-lapse X-ray video of pupation in six specimens of *Calliphora vicina*, beginning at 28 hours after pupariation (00:00, h:min). Images are displayed at one frame per second, each image captured at intervals of two-minutes (i.e. 120 x natural speed). The puparia on the top row (A-C) were imaged with the dorsal surface uppermost (specimen B inclined slightly to the left) while the puparia on the bottom row (D-F) were imaged with their left side uppermost. All puparia have their head at the top of the video. See text and Figure 1 for further explanation. Note that the gas bubble in specimen D has already started to move from the pupa at the start of the video and that the process starts next in specimen E then sequentially in specimens B, C, A and finally in F.

**Movies S3**

Stack of micro-CT based virtual sagittal sections of *Calliphora vicina* puparium at six hours after pupariation, scanned from left surface to right surface, dorsal surface on right-hand side of video, head at top. See text and Figure 2B for further explanation. Note the indentation in the cuticle of the puparium on the dorsal surface of the anterior end.

**Movies S4**

Stack of micro-CT based virtual horizontal sections of *Calliphora vicina* puparium at six hours after pupariation, scanned from dorsal surface to ventral surface, head at top. See text and Figure 2D for further explanation. Note the indentation in the cuticle of the puparium on the dorsal surface of the anterior end.

**Movies S5**

Stack of micro-CT based virtual sagittal sections of *Calliphora vicina* puparium at 24 hours after pupariation, scanned from left surface to right surface, dorsal surface on right-hand side of video, head at top. See text and Figure 2F for further explanation.

**Movies S6**

Stack of micro-CT based virtual horizontal sections of *Calliphora vicina* puparium at 24 hours after pupariation, scanned from dorsal surface to ventral surface, head at top. See text and Figure 2H for further explanation.

**Movies S7**

Stack of micro-CT based virtual sagittal sections of *Calliphora vicina* puparium at 30 hours after pupariation, scanned from left surface to right surface, dorsal surface on right-hand side of video, head at top. See text and Figure 2J for further explanation.

**Movies S8**

Stack of micro-CT based virtual horizontal sections of *Calliphora vicina* puparium at 30 hours after pupariation, scanned from dorsal surface to ventral surface, head at top. See text and Figure 2L for further explanation.
